# Priorization of River Restoration by Coupling Soil and Water Assessment Tool (SWAT) and Support Vector Machine (SVM) Models in the Taizi River Basin, Northern China

**DOI:** 10.3390/ijerph15102090

**Published:** 2018-09-23

**Authors:** Juntao Fan, Mengdi Li, Fen Guo, Zhenguang Yan, Xin Zheng, Yuan Zhang, Zongxue Xu, Fengchang Wu

**Affiliations:** 1 State Key Laboratory of Environmental Criteria and Risk Assessment, Chinese Research Academy of Environmental Sciences, Beijing 100012, China; fanjt@craes.org.cn (J.F.); limd@craes.org.cn (M.L.); guofenstephanie@gmail.com (F.G.); zgyan@craes.org.cn (Z.Y.); zhengxin@craes.org.cn (X.Z.); wufengchang@vip.skleg.cn (F.W.); 2 College of Water Sciences, Beijing Normal University, Beijing 100875, China; zxxu@bnu.edu.cn

**Keywords:** aquatic organisms, SWAT, SVM, river restoration, hydrological periods

## Abstract

Identifying priority zones for river restoration is important for biodiversity conservation and catchment management. However, limited data due to the difficulty of field collection has led to research to better understand the ecological status within a catchment and develop a targeted planning strategy for river restoration. To address this need, coupling hydrological and machine learning models were constructed to identify priority zones for river restoration based on a dataset of aquatic organisms (i.e., algae, macroinvertebrates, and fish) and physicochemical indicators that were collected from 130 sites in September 2014 in the Taizi River, northern China. A process-based model soil and water assessment tool (SWAT) was developed to model the temporal-spatial variations in environmental indicators. A support vector machine (SVM) model was applied to explore the relationships between aquatic organisms and environmental indicators. Biological indices among different hydrological periods were simulated by coupling SWAT and SVM models. Results indicated that aquatic biological indices and physicochemical indicators exhibited apparent temporal and spatial patterns, and those patterns were more evident in the upper reaches compared to the lower reaches. The ecological status of the Taizi River was better in the flood season than that in the dry season. Priority zones were identified for different hydrological seasons by setting the target values for ecological restoration based on biota organisms, and the results suggest that hydrological conditions significantly influenced restoration prioritization over other environmental parameters. Our approach could be applied in other seasonal river ecosystems to provide important preferences for river restoration.

## 1. Introduction

The ecological status of rivers is tightly related to human society. Human activities referring to industry, agriculture, and construction may affect important ecological functions and processes, such as nutrient cycling and carbon flux in food webs [1,2,3], change hydrological regimes, and lead to habitat degradation. The response of river ecosystems to those human activities varies with temporal and spatial scales, which poses a conundrum for river remediation and flow regulation [4,5]. There is a great interest in understanding how the ecological status of rivers changes with temporal and spatial scales, and identifying the priority zones of river ecosystems in order for effective catchment management and river conservation [6,7].

Previous studies mainly focused on the identification of rivers with hydromorphological or physicochemical restoration priority [8], whereas few considered the biological restoration as the priority. Aquatic organisms, i.e., algae, macroinvertebrates and fish, play an important role in river ecosystems [9], and they reflect the long-term cumulative effects of environmental pressure on river ecosystems [10,11]. In the United States, European Union, and Australia, aquatic organisms have been widely used to assess the health status and restoration effects on rivers and streams [12,13,14]. In China, scientists have pointed out that in face of temporal and spatial variations in rivers, aquatic organisms could provide useful information to evaluate the biological status of river ecosystems [3]. However, limited dataset of aquatic organisms and environmental pressure have led to a bottleneck of identifying the priority zones of river ecosystems.

Aquatic biological indices are directly related to physicochemical indicators, which are affected by the driving forces of watersheds, e.g., geography, climate, and human disturbance. To model aquatic physicochemical indicators, process-based hydrological models are becoming a popular approach to study the temporal and spatial dynamics of river ecosystems [15]. The modelling can provide intact hydrologic regime information, such as watershed runoff and sediment load, and reveal the inflowing process of the non-point source pollutants, such as nutrients and pesticide. Among those models, the soil and water assessment tool (SWAT) model that was developed by the USDA-ARS (U.S. Department of Agriculture, Agricultural Research Service) is considered to be a distributed hydrological model, and is suitable for long-term simulation of non-point source pollution in watersheds [16,17].

The mechanism on how aquatic organisms vary with space and time is complicated because of the synergistic effects of various environmental factors, e.g., hydrologic regimes and water quality. Therefore, it is important to accurately simulate the relationships between aquatic organisms and physicochemical factors [18]. A data-driven approach is necessary to correlate the bio-indicators and the observed physicochemical data. Support vector machine (SVM) model is able to interpret high-dimensional and high-nonlinear relationships, which is valuable for ecological research and management, and it has been applied in the prediction of spatial distribution of soil organic carbon [19] and chlorophyll-a [20]. The objective of this study is to construct a relationship between aquatic organisms and water physicochemical factors using appropriate models, and to achieve continuous simulation of aquatic organisms on a spatio-temporal scale through a distributed hydrological model. The construction and coupling of different models could make up the shortage of field monitoring data, comprehensively reflect the temporal-spatial dynamics of ecological status, and help in identifying priority areas of river restoration in watersheds. 

## 2. Methods

### 2.1. The Framework of Model Coupling 

**Figure 1:**
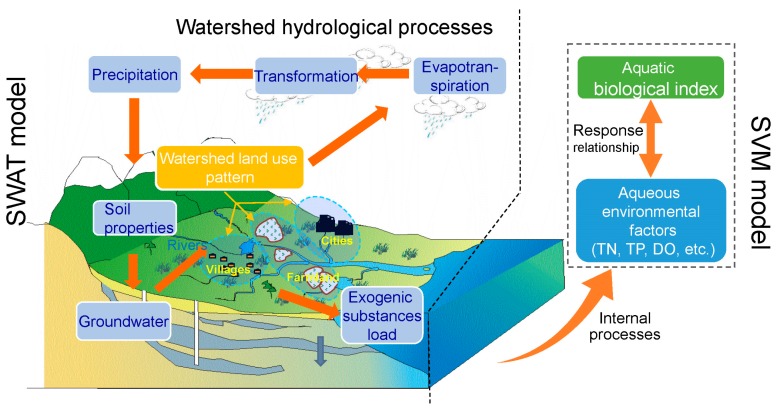
Conceptual diagram of SWAT (soil and water assessment tool) and SVM (support vector machine) model coupling.

The generation and migration of non-point source pollution in the basin is mainly determined by three major processes: surface rainfall-runoff process, rainfall-runoff erosion process, and leaching of soil by surface, soil, and underground runoff. Subsequently, the pollutants enter the water body, affecting the distribution of physicochemical indicators of the water body (Figure 1). The SWAT model, which was based on physical process to simulate the flow environment and explore the temporal-spatial dynamics of physicochemical indicators, was applied at the watershed-scale in the Taizi River in northern China. Moreover, the SVM model was employed to analyze the response of aquatic organisms to environmental factors. The SWAT modelling results were coupled with SVM modelling inputs in order to simulate the temporal-spatial distribution of aquatic organisms, and the result was further validated by the measured data (Figure 1).

### 2.2. Study Area

**Figure 2:**
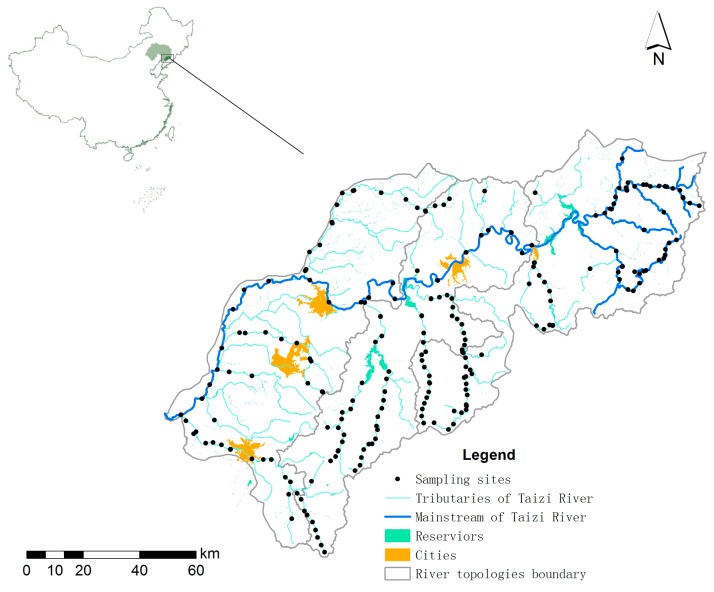
Sampling locations in the Taizi River Basin, northern China.

The Taizi River is located in the southeast of Liaoning Province, northern China (122°23’ E–122°53’ E, 40°28’ N–41°39’ N, Figure 2). It flows through Benxi City, Liaoyang City, Anshan City, and Haicheng City, and it covers a basin area of 13,900 km^2^, with a length of 413 km. Within the warm temperate sub-humid area, the Taizi River Basin has a continental monsoon climate. The upper reach of the basin is characterized by low hilly landform, whereas most river channels are between the valleys, with relatively less human exploration and more vegetation coverage. Many rare aquatic organisms have been recorded in this region, such as clean-type fishes (*Lampetra morii*, *Odontobutis Obscurus*, etc.), and clean large-scale macrobenthos (*Epeorus melli*, *Cambaroides dauricus*). In contrast, the middle and lower reaches are the plain area, with the terrain of higher southeast and lower northwest. Meandering river channels represent a curved type river. More distributed industries and human disturbance have led to excessive land utilization in lower reaches. In recent years, urbanization results in increasing pressure on the Taizi River Basin, such as water quality deterioration, habitat degradation, and biodiversity loss [21,22]. 

### 2.3. Field Sampling and Indicator Selection

The dataset was obtained from field investigation in September 2014, including 130 sampling sites along the main channels and tributaries of the Taizi River (Figure 2) [21,23]. Biological communities (i.e., fish, algae, and macroinvertebrates), and environmental parameters (i.e., dissolved oxygen (DO), electricity conductivity (EC), ammonia nitrogen (NH_3_-N), chemical oxygen demand (COD), biological oxygen demand in five days (BOD_5_), total phosphorus (TP), total nitrogen (TN), and water quantity (WQ)) were sampled.

Fish samples were collected by electronic fishing and gill net fishing, and all fish samples were identified, enumerated, and weighed in situ. Rare or unknown species were preserved with 4% formalin for identification in the laboratory. Benthic macroinvertebrates were collected using a Surber net (30 cm × 30 cm, 500 μm mesh) and D-frame dip net (15 cm radius and 500 μm mesh), and they were identified to the genera level in the laboratory. Benthic algae were collected from all available substrates and habitats at each site, and were identified to the species level in the laboratory. Physicochemical parameters were measured in situ (i.e., DO and EC) or determined from water samples in the laboratory (i.e., NH_3_-N, COD, BOD_5_, TP, and TN), according to the Chinese Water Quality Standard Methods [24].

Eight biological indicators were implemented in the SVM model, i.e., fish species richness (F_S), fish index of biotic integrity (F_IBI), fish Berger-Parker index (F_BP), macroinvertebrate families richness (M_S), biological monitoring working party (M_BMWP), *ephemeroptera*, *plecoptera* and *trichoptera* family richness (M_EPT), algae species richness (A_S), and algae Berger-Parker index (A_BP). Among these indicators, F_S, F_IBI, and F_BP are related to physical, chemical, biological and zoogeographic factors, and long-term pressures [21]. M_S is a measure of diversity of macroinvertebrates, which reflects the general deterioration of water quality [25]. M_BMWP is used to assess organic pollution in freshwaters [26]. M_EPT is the taxa richness within the insect group, which is sensitive to contamination [27]. A_S and A_BP both reflect the water quality deterioration related to eutrophication and organic pollution [21]. These indicators were listed in Table 1, together with the related impact typologies.

### 2.4. SWAT Modelling

The digital elevation map (DEM), land use, soil type, meteorological station data, reservoir data, and agricultural production data (Table 2) were input in the SWAT model. Modelling results were integrated in the Access database, which could be displayed in ArcGIS 10.1 Version (Esri, Redlands, CA, USA).

In this study, the Taizi River Basin was divided into 130 sub-basins, and the sampling locations were used as the outlets in ArcSWAT ArcGIS extension 2012 Version (Blackland Research and Extension Center, Texas Agrilife Research & Grassland, Soil and Water Research Laboratory USDA Agriculture Research Service, Texas, USA). Data on hydrological stations, reservoirs, point emissions, and agricultural management information were also loaded for SWAT modelling. Three typical hydrological years were selected to investigate the distribution of aqueous environment factors in each sub-basin, i.e., flood year (2012), average water year (2004), and dry year (2014). The typical months of the dry season (April), the flood season (September), and the average water season (November) were chosen for modelling from the above three years. Therefore, the distribution of five environmental factors of WQ, TP, TN, DO, and BOD_5_ was explored in nine different periods. Values of aqueous environment factors were calculated by the SWAT model output file (*rch* file).

It is necessary to calibrate the sensitive parameters from the modelling results, as there are more than 1000 parameters in SWAT, which can greatly improve the efficiency of the model. In this study, the SWAT-CUP toolbox, which is based on a mathematical algorithm shuffled complex evolution (SCE-UA) from the research of the University of Arizona, was used to automatically determine the parameters. SCE-UA is generally considered the most efficient and effective method [28], and is widely applied in the parameter calibration of hydrological models and other aspects, such as soil erosion, groundwater, remote sensing, and surface water simulation. In our study, the runoff parameters were calibrated from 1980 to 1992, and verified from 1992 to 2002, the physiochemical parameters were calibrated from 2007 to 2008, and verified from 2009, at a monthly scale. Nash-Sutcliffe coefficient (NS) and the coefficient of determination (R^2^) were adopted as indicators to evaluate the calibration results. NS demonstrated the ratio of the residual variance to the variance of the measured data [29], showing the comparison of the ratio of observed value to simulated value with the 1:1 line. NS values ranged from 0 to 1. If the value is close to 1, then it indicates better modelling results are required; if NS ≥ 0.5, the results can be accepted. For R^2^ values, if it is close to 1, it suggests that better modelling results are required. If R^2^ is ≥0.6, the results can be accepted.

### 2.5. SVM Modelling

The SVM is a kernel-based learning algorithm, and it is widely used for pattern classification and regression [30]. In this study, 10 training and validation subsets were built. In each subset, 90% samples were used for training and 10% were for validation. Various search algorithms were applied to determine optimal parameters for the SVM model based on the lower values of the root-mean-square error (MSE) in the validation subset. The squared correlation coefficient (R^2^) was chosen to describe the overall modelling performance.

A sensitivity analysis was applied to investigate sensitive environmental parameters that influence the response of biological indices. The one-factor-at-a-time (OAT) method was used as the assessment tool for checking sensitivity of model variables. The SVM models were running by removing a variable at a time with other parameters being constant. The variation in overall model performance (squared correlation coefficient, R^2^) for a given variable was subsequently calculated to obtain the effects of the variable on the model performance, and this process was repeated for every variable. At this stage, the biological indices were selected for the simulation of their temporal-spatial dynamics with the aid of the SWAT model.

### 2.6. Identification of the Priority Areas 

The priority sub-basins in different hydrological periods were identified by setting target values of ecological restoration. Firstly, three watershed-scale habitat typologies, i.e., highlands, midlands and lowlands, were taken from previous studies in the Taizi River Basin. Secondly, these typologies were used to establish target values for selected indicators. For the highlands, F_S was ‘good’, and DO, TN, and TP should meet the level ‘II’ of Surface Water Environmental Quality Standards of China (GB3838-2002) [31]. For the midlands and lowlands, F_S should reach the ‘general’ level, and DO, TN, TP should meet the level ‘IV’ of GB3838-2002. The specific value of each index was shown in Table 3. The target values for F_S were derived from expert opinion.

## 3. Results and Discussion

### 3.1. Responses of Aquatic Biological Indices to Phsicochemical Indicators

**Figure 3:**
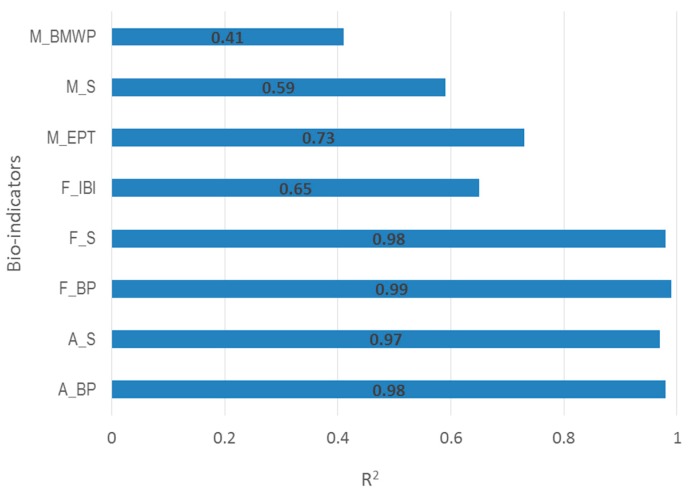
Squared correlation coefficient (R^2^) values for SVM (support vector machine) models performance.

R^2^ values for each different SVM model were shown in Figure 3. All of those models achieved high values of explained variance (R^2^ > 0.6) except M_BMWP and M_S, which were 0.41 and 0.59, respectively. The result indicated that the indices of fish communities (i.e., F_BP, F_S) and algal communities (i.e., A_BP, A_S) were better fitted with the environmental variables when compared with the indicators of macroinvertebrate fauna (i.e., M_BMWP, M_S). Therefore, the indices of fish and algal communities were selected to simulate their temporal-spatial dynamics.

Further, our results showed that, in the Taizi River, the SVM model could be a reliable prediction tool for fish and algal communities based on selected environmental factors. However, the ability of the model to predict macroinvertebrate communities was limited, indicating the increased number of pollution tolerance species (i.e., *Orthocladiinae*, *Oligochaeta*), and a reduced sensitivity to environmental stress in the Taizi River Basin. 

Agricultural activities were the major type of human disturbance in this area, and significantly affected algal communities. Hydrological status (e.g., water quantity) and physiochemical conditions (e.g., COD, EC, TN) were both considered in the SVM, and played a crucial role in the reproduction and predation of fish communities [32].

Table 4 showed the R^2^ for every input variable in the SVM model. OAT analysis checked the model fitting by removing a variable. If R^2^ became smaller, it suggests a greater impact on the model fit, and the variable was more sensitive. For algal communities, the smallest R^2^ for A_BP was 0.94 (TP), and for A_S was 0.90 (TN). For fish communities, R^2^ for F_BP was 0.93 (BOD_5_), for F_IBI was 0.62 (DO), and for F_S was 0.93 (BOD_5_). For macroinvertebrate communities, R^2^ for M_BMWP was 0.35 (BOD_5_), for M_EPT was 0.65 (TN), and for M_S was 0.54 (TP). The result suggests that these sensitive environmental indicators were appropriate for the SWAT model.

Sensitivity analysis of the SVM model showed that algae and macroinvertebrates were more sensitive to nutrients, whereas fish communities were more sensitive to DO and organic pollutants. It has been documented that nutrients was a limiting factor for algal and macroinvertebrate communities [33]. Low levels of DO posed an impact on the tolerance limit of fish [34], affecting the structure of fish communities. In the marine environment, many fish became stressed at a DO level of 4.5 mg/L [35]. In the Taizi River, it has been reported that DO and other physicochemical indicators (such as TN and pH) had significant effects on fish spatial distribution at reach scale [36].

### 3.2. Temporal-Spatial Variations in Phsicochemical Indicators

The statistical indices (Table 5) showed that R^2^ and NS in calibration periods of each hydrological station were higher than 0.7. R^2^ in validation periods was higher than 0.6, and NS in validation periods was higher than 0.7.

**Figure 4:**
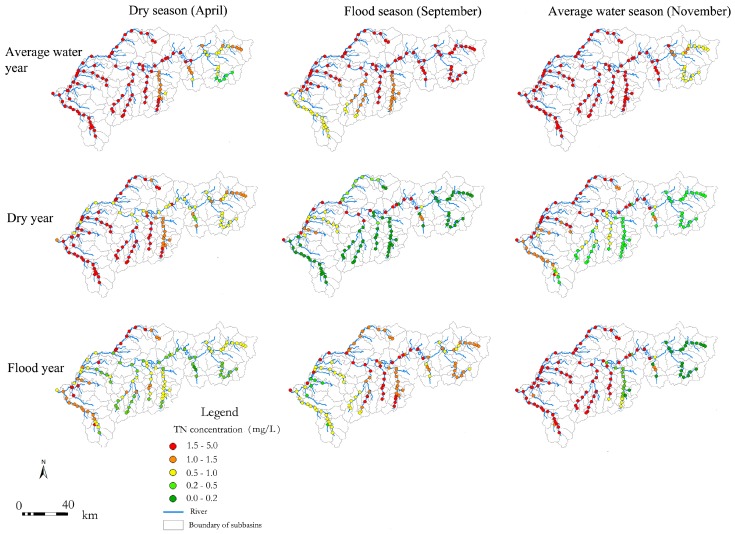
Temporal and spatial dynamics of TN concentration.

After calibration, the SWAT model was used to simulate the temporal-spatial variations in TP, TN, DO, and BOD_5_ concentrations. Results demonstrated that aqueous environment factors displayed apparent spatial and temporal patterns. Spatially, TP, TN, DO, and BOD_5_ concentrations exacerbated gradually from upstream to downstream, and they were generally lower in tributaries than the mainstream, which was consistent with human disturbance gradient. Temporally, water quality in the flood season was better than that in the dry season and the average water season (Figure 4, taking TN as an example).

**Figure 5:**
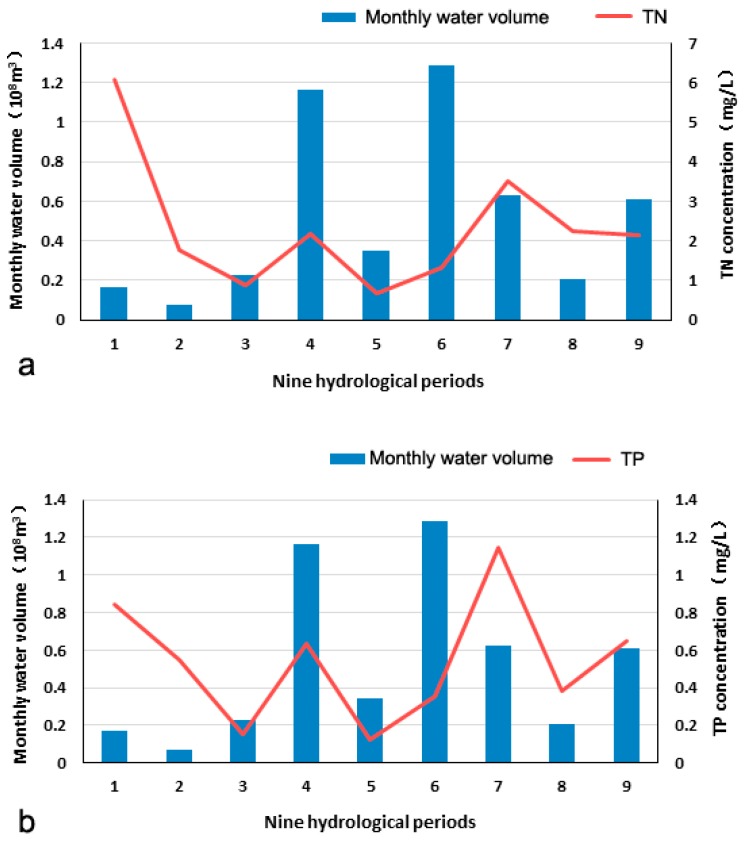
Relationships between monthly water volume and TN (**a**) and total phosphorus (TP) (**b**).

However, the temporal distribution of physicochemical indicators was not completely consistent with the annual and seasonal water flow variations. The correlation coefficients between TN/TP concentration and water flow were −0.19 and −0.10, respectively (Figure 5). The relationship between pollutant concentrations and water flow was unclear. The modelling result indicated that hydrological characteristics had an effect on pollutant inputs in rivers, whereas the distribution of the physical and chemical indicators in water bodies was more likely related to the intensity of human activities.

### 3.3. Temporal-Spatial Dynamics of Aquatic Organisms

**Figure 6:**
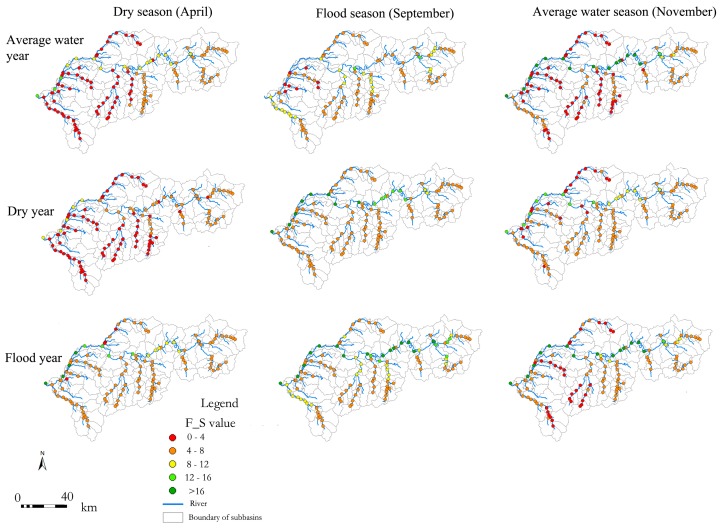
Temporal-spatial distribution dynamics of Fish Species Richness (F_S).

A fish ecological index (F_S, R^2^ = 0.98) and an algal ecological index (A_S, R^2^ = 0.97) were selected to simulate the temporal-spatial dynamics by coupling SVM and SWAT models. F_S exhibited an obvious deterioration from upstream to downstream, whereas those from the upper and middle reaches were greater than those from the tributaries, and those of the tributaries from upper headstream were greater than those in the middle and lower reaches. Further, F_S displayed the greatest value in the flood year, followed by that in the dry year, whereas those in the average water year were the lowest. Additionally, F_S showed the greatest value in September, while those from November and April were relatively lower (Figure 6).

**Figure 7:**
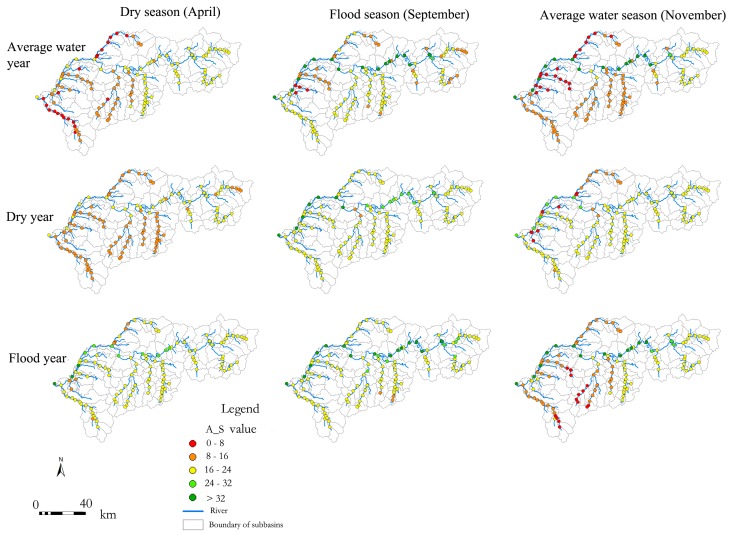
Temporal-spatial distribution of Algae Species Richness (A_S).

The spatial variation in A_S was similar to that of F_S. A_S gradually decreased from upstream to downstream, and it was greater in upper and middle reaches than the tributaries. A_S was greater in tributaries from upper headstream than those of middle and lower reaches, which was more apparent in the low water season than in the flood season. For annual variations, the greatest A_S appeared in the flood year, whereas the lowest was in the average water year. For monthly variations, the greatest A_S was in September, whereas November and April had relatively lower A_S values (Figure 7).

**Figure 8:**
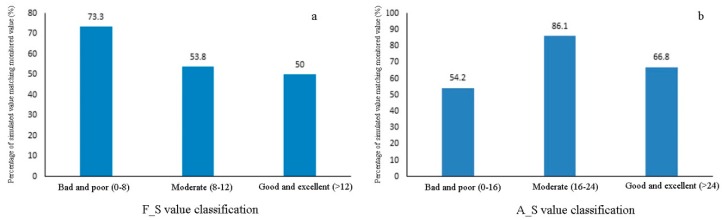
Verification of F_S (**a**) and A_S (**b**) simulation results.

The F_S and the A_S were validated in September 2014 with monitored results (Figure 8). According to the results, F_S in the level ‘poor and very poor’ (0–8) had an overlap ratio of 73.3%, whereas those in the level ‘general’ (8–12) had an overlap ratio 53.8%, and for the level ‘good and very good’ (>12) the overlap ratio was 50%. A_S in the level ‘poor and very poor’ (0–16) had an overlap ratio of 54.2%, whereas those that were in the level ‘general’ (16–24) had an overlap ratio 86.1%, and for the level ‘good and very good’ (>24) the overlap ratio was 66.8%. The simulation results of F_S and the measured values showed the highest overlap ratio in the level ‘poor and very poor’, indicating that simulated values were generally lower than measured values. This may be attributed to that measured values were affected by sampling methods, time, and other random factors, which may induce a wider range than simulated values. As for the A_S, the highest overlap ratio appeared in the level ‘general’, suggesting that simulated values were more approximate to the measured values.

The response of aquatic communities to environmental factors is very complex, not only to pollution in water bodies, but also to habitat physical conditions [37]. Previous studies pointed out that the amount of water is closely related to habitat status, and the quantitative relationship between flow and habitat indicators can be established through a certain relationship [38]. Therefore, the change of water quantity has an impact on the habitat of aquatic communities.

### 3.4. Identification of the Priority Areas in Different Hydrological Periods

**Figure 9:**
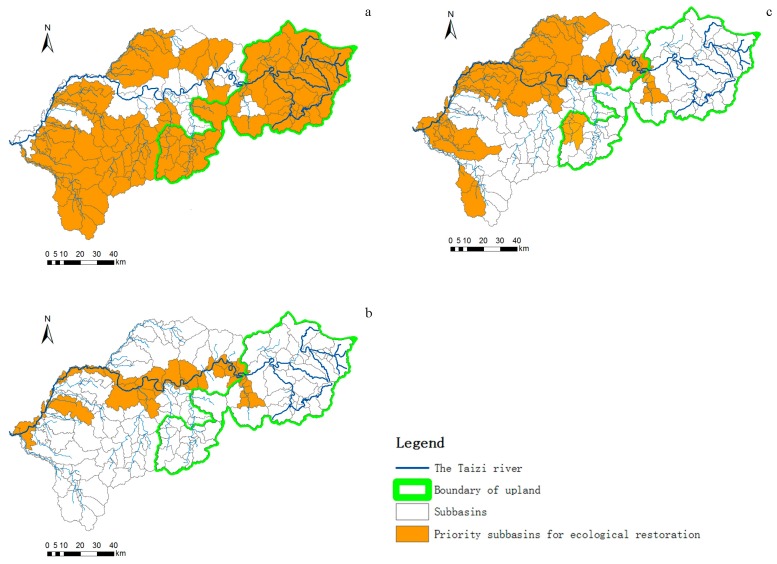
Priority zones (sub-basins) for ecological restoration in the dry season (**a**), the flood season (**b**), and the average water season (**c**).

Figure 9a showed that most sub-basins needed restoration in the dry season to meet the requirement of target values set in Table 3. In the upstream area, most of the sub-basins needed rehabilitation. In the middle and lower reaches, the majority of sub-basins of tributaries required restoration, whereas only several sub-basins of the mainstream were not demanding urgent rehabilitation. In contrast to the dry season, less sub-basins required rehabilitation in the flood season (Figure 9b). In the upstream area, only the sub-basin of the downstream tributary in Xiaotang River in Benxi County needed to be repaired, whereas in the middle and lower reaches, sub-basins, which required restoration, were mainly located along the mainstream. The number of sub-basins requiring rehabilitation in the average water season were less than that in the dry season (Figure 9c), but more than that in the flood season. In the upper reaches, the sub-basins of the tributary flowing from Guanyinge Reservoir through the Nandianzi Town of Benxi County, and the tributary sub-basin of the Nanfen District of Benxi City needed to be repaired. In the middle and lower reaches, sub-basins along the mainstream and the northeastern part of the North Shahe River tributaries demanded restoration, whereas most sub-basins in the south, except the Haicheng River tributaries, showed relatively better status with no need for restoration.

The result showed that the number of priority zones for ecological restoration was tightly related to hydrological characteristics within the watershed. More sub-watersheds need to be repaired in the dry season, followed by the average water season and the flood season, indicating that aquatic biodiversity decreased as the water quantity declined. Former studies have demonstrated strong correlations between water ecological status and water quantity, which is consistent with our result. Accordingly, river restoration mainly concentrated on water quantity recovery [8]. A number of techniques for riverine restoration have been operated to address the hydrological problems, for example, water diversion and constructed wetland. The former dilutes and transports contaminants by importing a large volume of clean water from elsewhere which has better water quality, and the latter allows for the river to maintain a certain amount of water during the dry season [39]. The modelling results suggest that identifying the ecological restoration priority zones by the aquatic ecological data from one hydrological period is not completely reliable, as the ecological status varies with the hydrological characteristics. Therefore, the priority zones of river ecosystems with different hydrological characteristics should be considered to acquire comprehensive information.

## 4. Conclusions

In this study, a method of temporal-spatial dynamic modelling of aquatic ecological status for ecological restoration by coupling SWAT and SVM models was established in the Taizi River, China. Results showed that there were significant temporal-spatial variations in physicochemical factors (TP, TN, DO, and BOD_5_) and aquatic biological indices (F_S and A_S). From upstream to downstream, physicochemical indicators displayed a gradual deterioration, whereas the upper and middle reaches of the mainstream showed better status than tributaries. Moreover, results indicated that tributaries from the upper reaches were characterized by greater quality than those from the middle and lower reaches. Further, aquatic organisms and aqueous physicochemical indicators implied the best ecological status in the flood season, and the worst in the dry season. Simulated values of aquatic organism indices were in good agreement with the measured values. Based on aqueous ecological dynamics, the priority zones of river ecological restoration in watersheds were identified. The results demonstrated that the sub-basin of the tributary flowing from Guanyinge Reservoir through Nandianzi Town of Benxi County was the key area of ecological restoration. The remedial priority area varied with hydrological seasons in the middle and lower reaches. More sub-basins required restoration in the dry season and less in the flood season. The approach that is proposed in this study could provide references for the decision-making of the ecological restoration strategy for other river ecosystems.

## Figures and Tables

**Table 1:** Indicators applied in the SVM (support vector machine) model and related impact typologies.

Indicators for the Ecological Status		Impact Typologies
Biological indicators		
Fish	Species Richness (F_S)	General degradation
	Index of Biotic Integrity (F_IBI)	General degradation
	Berger-Parker Index (F_BP)	General degradation
Macroinvert-ebrate	Families Richness (M_S)	General degradation
	BiologicalMonitoring Working Party Score (M_BMWP)	Organic pollution
	Ephemeroptera, Plecoptera and Trichoptera Family Richness (M_EPT)	General degradation
Algae	Species Richness (A_S)	General degradation
	Berger-Parker Index (A_BP)	General degradation
Physicochemical indicators		
Electric Conductivity (EC)		Salinization
Dissolve Oxygen (DO)		Organic pollution
Biological Oxygen Demand in 5 days (BOD_5_)		Organic pollution
Chemical Oxygen Demand (COD)		Organic pollution
Ammonia Nitrogen (NH_3_-N)		Eutrophication
Total Phosphorus (TP)		Eutrophication
Water Quantity (WQ)		Alteration of hydrological regime

**Table 2:** Geography, climate and agricultural data used for the SWAT model.

Data Type	Category	Description	Data Source
Geographic information	DEM	30 m × 30 m	SRTM DEM
	Land use map	Land use type patterns (1:100,000)	Spot image interpretation
	Soil type map	Soil type patterns	Institute of Soil Science, Chinese Academy of Sciences
Meteorological information	Meteorological data	Meteorological factor daily data	China Meteorological Administration
Rainwater information	Precipitation	Daily precipitation data (1979–2015)	Liaoning Institute of Water Resources and Hydropower Research
	Hydrological Information	Basic station information and daily hydrological data (1978–2002)	Liaoning Institute of Water Resources and Hydropower Research
	Reservoir information	Eigenvalues, releasing water	Liaoning Institute of Water Resources and Hydropower Research
Point source information	Information of sewage inlets to the river	Location, blowdown, emission volume, TN, TP, COD and NH_3_-N	Liaoning Institute of Water Resources and Hydropower Research
Agricultural management information	Agricultural management measures	The type of crop and fertilization information	Investigation data, Statistical Yearbook, literatures, etc.

SRTM: Shuttle Radar Topography Mission; DEM: digital elevation map.

**Table 3:** Target values for ecological restoration in the Taizi River.

Habitat Typologies	Target Values			
	F_S	DO (mg/L)	TN (mg/L)	TP (mg/L)
Highlands	≥12	≥6	≤0.5	≤0.1
Midlands and lowlands	≥8	≥3	≤1.5	≤0.3

**Table 4:** Squared correlation coefficient (R^2^) values for sensitivity analysis.

Variables	EC	DO	BOD_5_	COD	NH_3_-N	TP	TN
A_BP	0.98	0.96	0.96	0.97	0.97	0.94	0.98
A_S	0.96	0.92	0.95	0.96	0.95	0.93	0.90
F_BP	0.97	0.94	0.93	0.98	0.97	0.95	0.94
F_IBI	0.65	0.62	0.63	0.65	0.64	0.63	0.63
F_S	0.96	0.94	0.93	0.96	0.97	0.98	0.96
M_BMWP	0.40	0.39	0.35	0.36	0.41	0.38	0.39
M_EPT	0.69	0.67	0.66	0.66	0.71	0.67	0.69
M_S	0.57	0.55	0.58	0.58	0.57	0.54	0.56

**Table 5:** Model performance statistics of the simulated and measured runoff and total nitrogen (TN) during calibration and validation.

Runoff	Hydrological Station	Measured Value (m^3^/s)	Simulated Value (m^3^/s)	R^2^	NS
Calibration period (1980–1992)	Benxi	41.647	39.842	0.73	0.71
	Liaoyang	54.108	53.047	0.81	0.83
	Xiaolinzi	66.442	67.271	0.82	0.83
	Tangmazhai	76.831	77.118	0.84	0.81
	Haicheng	4.493	4.329	0.78	0.82
Validation period (1993–2002)	Benxi	37.357	42.861	0.69	0.71
	Liaoyang	47.271	49.706	0.78	0.82
	Xiaolinzi	59.408	58.573	0.81	0.79
	Tangmazhai	70.266	71.586	0.82	0.80
	Haicheng	4.459	9.213	0.77	0.83
**TN**	**Hydrological Station**	**Measured Value (mg/L)**	**Simulated Value (mg/L)**	**R^2^**	**NS**
Calibration period (2007–2008)	Benxi	3.704	3.912	0.85	0.81
	Liaoyang	4.916	5.101	0.86	0.83
	Xiaolinzi	5.293	5.012	0.79	0.76
	Tangmazhai	8.600	7.896	0.70	0.72
Validation period (2009)	Benxi	3.206	2.963	0.82	0.80
	Liaoyang	5.126	4.858	0.78	0.76
	Xiaolinzi	6.211	7.213	0.69	0.70
	Tangmazhai	8.422	7.689	0.75	0.77
